# Multimodal Imaging in Retinal Vascular Occlusions following Trauma – A Case of Sickle Cell Disease with Negative Sickling Test

**DOI:** 10.18502/jovr.v16i3.9444

**Published:** 2021-07-29

**Authors:** Ekta Singh Sahu, Mani Sachdeva, Alok Sen, Samendra Karkhur

**Affiliations:** ^1^Vitreo Retina and Uvea Services, Sadguru Netra Chikitsalaya, Chitrakoot, India

##  PRESENTATION 

**Figure 1:**
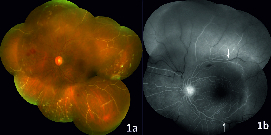
a. Colour fundus photograph of the left eye at first visit showing multiple retinal arteriolar and venous occlusions and few scattered retinal hemorrhages mid peripherally. b. Fluorescein angiography of the left eye at first visit shows absence of filling of dye in posterior and peripheral retina with hyperfluorescent column (marked with white arrow) at junction of abrupt obstruction of circulation.

**Figure 2:**
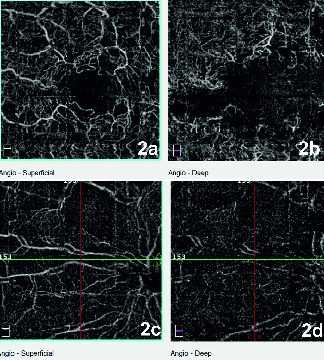
a. Colour fundus photograph of the left eye at first visit showing multiple retinal arteriolar and venous occlusions and few scattered retinal hemorrhages mid peripherally. b. Fluorescein angiography of the left eye at first visit shows absence of filling of dye in posterior and peripheral retina with hyperfluorescent column (marked with white arrow) at junction of abrupt obstruction of circulation.

**Figure 3:**
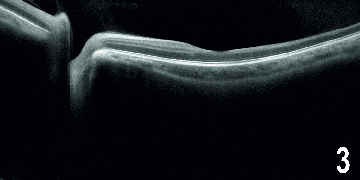
Optical coherence tomography at first visit demonstrate corresponding area of inner retinal thinning at macula temporally.

A 30-year-old male presented with diminution of vision in his left eye (1/60), intraocular pressure (IOP) of 40 mmHg and full anterior chamber hyphema, following a blunt trauma 15 days before. His right eye was normal. An anterior chamber washout was performed in his left eye following which his visual acuity improved to 6/12 and IOP reduced to 24 mm Hg. Postoperative funduscopy showed multiple retinal vascular chalky-white occlusions, with focal spasm of vessels and superficial retinal hemorrhages [Figure 1a]. Fluorescein Angiography (FA) showed abrupt obstruction of vascular flow, predominantly in the arterioles. Fragmentation and segregation of blood column appeared as hyperfluorescent plugs at the junction of perfused and non-perfused retina [Figure 1b]. Optical coherence tomography angiography (OCTA) showed ischemic change at the peripapillary and parafoveal capillary plexus [Figures 2a–2d], with enlargement of the foveal avascular zone, primarily in deeper plexus secondary to higher capillary dropout. The corresponding area on the OCT raster scan showed splaying of the macula with temporal thinning of the inner retina [Figure 3].

**Figure 4:**
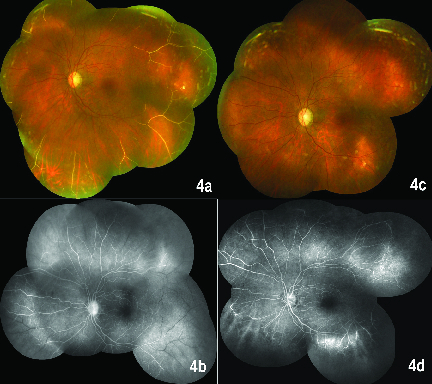
a. Colour fundus photograph of the left eye at 2 weeks follow up documents fragmentation of blood column into aggregations of erythrocytes in occluded network of vessels. b. Fluorescein angiography of the left eye at 2 weeks follow up outlines largely occluded peripheral vessels. c. Colour fundus photograph of the left eye at 4 months showing attenuation of previously occluded vessels. d. Fluorescein angiography of the left eye at 4 months follow up shows arterio-venous shunt and microaneurysms compensatory changes.

**Figure 5:**
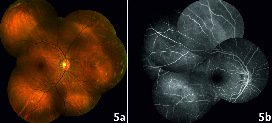
a,b. Colour fundus photograph and Fluorescein angiography of the right eye.

Blood examination suggested chronic anemia with reduced Hb (8.9%), MCV (79.9 fL), and MCHC (29.7 g/dl). The patient had been on supplements for anemia for six years although unaware of the underlying cause. However, upon noticing an abnormal gait and further inquiry, history of hip replacement surgery for obstructed blood supply was elicited. Underlying sickle cell disease (SCD) was strongly suspected but the “sickling test” was negative. Testing for hypercoagulable states, cardiac valvular disorder, and systemic vasculitis screening did not reveal the systemic cause of vascular occlusion. At a follow-up after two weeks and one month, fragmentation of blood column in the vessels [Figures 4a and 4b] and “silver wiring” appearance of occluded retinal vessels were noted. Ischemic maculopathy persisted on OCTA.

After four months, the patient shared his previous clinical records, that confirmed diagnosis of SCD with a “positive” sickling test and high-performance liquid chromatography (HPLC) testing had been advised. He was on oral hydroxyurea treatment. The natural course of the disease progressed in accordance with occlusive phenomenon showing partial remodeling of the vascular network with recanalization of vessels [Figure 4c]. A repeated FA revealed compensatory microaneurysms due to ischemic insult in the peripheral retina without signs of neovascularization [Figure 4d]; hence, we kept him under observation.

##  DISCUSSION

The peripheral vascular occlusion exhibited in SCD is caused by the sickling process. A hypoxic environment in the presence of hyphema incites a cycle where sickled erythrocytes increase the IOP, which in turn cause hypoperfusion. The terminal type of vascular architecture of the peripheral retina succumbs to hypoperfusion and precipitates vascular occlusion. Similar events occurred in our case and led to unilateral manifestation of retinopathy. While the FA contemplates the peripheral vascular abnormalities, sickle maculopathy may not be clinically apparent on funduscopy or detected on FA. However, it is well demonstrated on spectral domain OCT and OCTA.^[1,2]^ The precapillary arteriole occlusion at the macula causes infarction in inner retinal layers while the unaffected larger caliber choriocapillaris keeps the outer retinal layers intact.^[2]^ These subtle vascular changes of the macula on imaging can help detect an underlying etiology in cases of retinal vascular occlusion. Since the OCT prior to trauma was not available and the fellow eye was normal [Figures 5a and 5b], it remains debatable whether macular ischemia was precipitated or coincidentally unmasked by trauma. In this case, the sickling test was falsely negative, probably because the patient was on hydroxyurea treatment; this enhances fetal hemoglobin levels and prevents sickling.^[3]^ Currently HPLC is the recommended preliminary test for its superior reliability and reproducibility.^[4]^

Existing literature is limited on retinal features and associated imaging findings obtained acutely in a case of SCD, where trauma has precipitated an ischemic event. Problems in this case also arose as the negative sickling test confused the initial diagnosis. We suggest considering SCD-associated retinopathy in similar clinical presentations and subjecting such patients to multimodal imaging and more confirmatory diagnostic blood tests.

##  Declaration of Patient Consent

The authors certify that they have obtained all appropriate patient consent forms. In the form the patient has given his consent for his images and other clinical information to be reported in the journal. The patient understand that his name and initial will not be published and due efforts will be made to conceal his identity, but anonymity cannot be guaranteed.

##  Financial Support and Sponsorship

Nil.

##  Conflicts of Interest

There are no conflicts of interest.
